# Prevalence and associated risk factors of human intestinal parasitic infections: a population-based study in the southeast of Kerman province, southeastern Iran

**DOI:** 10.1186/s12879-019-4730-8

**Published:** 2020-01-06

**Authors:** Mohammad Javad Abbaszadeh Afshar, Maryam Barkhori Mehni, Mostafa Rezaeian, Mehdi Mohebali, Vali Baigi, Somayeh Amiri, Mohammad Bagher Amirshekari, Ruhollah Hamidinia, Mohammad Samimi

**Affiliations:** 1 Department of Medical Parasitology and Mycology, School of Public Health, Tehran University of Medical Sciences, Tehran, Iran; 2 Health Affairs, Jiroft University of Medical Sciences, Jiroft, Iran; 3 Sina Trauma and Surgery Research Center, Tehran University of Medical Sciences, Tehran, Iran

**Keywords:** Intestinal parasites, Human, Prevalence, Iran

## Abstract

**Background:**

Determination of the prevalence and distribution pattern of intestinal parasites is a fundamental step to set up an effective control program to improve the health status. This study aimed to determine the prevalence of intestinal parasitic infections and associated risk factors among inhabitants of Rudbar-e Jonub county, southeast of Kerman province, southeastern Iran.

**Methods:**

In this cross-sectional study, 861 stool specimens were collected from inhabitants of Rudbar-e Jonub county through a multistage cluster sampling method in 2018. The collected specimens were examined by parasitological methods including, direct wet-mounting (for the fresh specimens with a watery consistency), formalin-ethyl acetate sedimentation and agar plate culture.

**Results:**

The prevalence of intestinal parasites was 34.2% (95% CI 30.1 to 38.2). The prevalence of protozoan parasites 32.3% (95% CI 28.4 to 36.5) was significantly higher than helminthic parasites 3.2% (95% CI 2.1 to 4.7). *Blastocystis sp.* (13.3%), *Entamoeba coli* (11.4%) and *Giardia lamblia* (10.6%) as protozoan parasite and *Hymenolepis nana* (2.4%) as helminthic parasite were the most common detected intestinal parasites in the study. *Entamoeba histolytica/dispar* (1.5%), *Iodamoeba bütschlii* (1.0%), *Chilomastix mesnili* (0.5%), *Entamoeba hartmanni* (0.4%), *Enterobius vermicularis* (0.3%) and *Ascaris lambercoides* (0.3%) were other detected parasites. Multiple logistic regression revealed a significant association of intestinal parasitic infections with source of drinking water and residency status (rural/urban). Multiple infections with 2 or 3 parasitic agents constituted 22.7% of 295 infected cases.

**Conclusions:**

This study revealed a high prevalence of intestinal protozoan infections among inhabitants of Rudbar-e Jonub county. Intestinal parasites especially protozoans remain a challenging public health problem wherever sanitation and health measures are limited in Iran.

## Background

Despite the advancement in sanitation infrastructure and hygiene status, intestinal parasitic infection remains a considerable public health problem, especially in developing countries [1]. It is estimated that more than three billion people (mostly children) are infected with intestinal parasites around the world [1, 2]. In Iran, due to diversity in socioeconomic, geographic, sanitary/hygiene, cultural, and educational status a broad range of intestinal parasites prevalence between 4.7 to 56% have been reported in the apparently healthy populations [3, 4]. Because of low socioeconomic status, limited sanitation, and also geographic factors, rural areas are regarded as endemic areas of intestinal parasitic infections in the southern part of Iran [5, 6].

Determination of the prevalence and distribution pattern of intestinal parasitic infections is a fundamental step to set up a prevention and control program to improve the health status. On the other hand, due to diversity in geographic factors and socio-cultural patterns in different parts of Iran, an epidemiological study is required in each region separately. To our knowledge, there is no study available on the distribution of intestinal parasitic infections in Rudbar-e Jonub county as a tropical area and with a deprived community in southeast of Kerman province, southeastern Iran. Therefore, this study conducted to determine the prevalence and risk factors associated with intestinal parasitic infections in Rudbar-e Jonub county inhabitants.

## Methods

### Study area

**Fig. 1:**
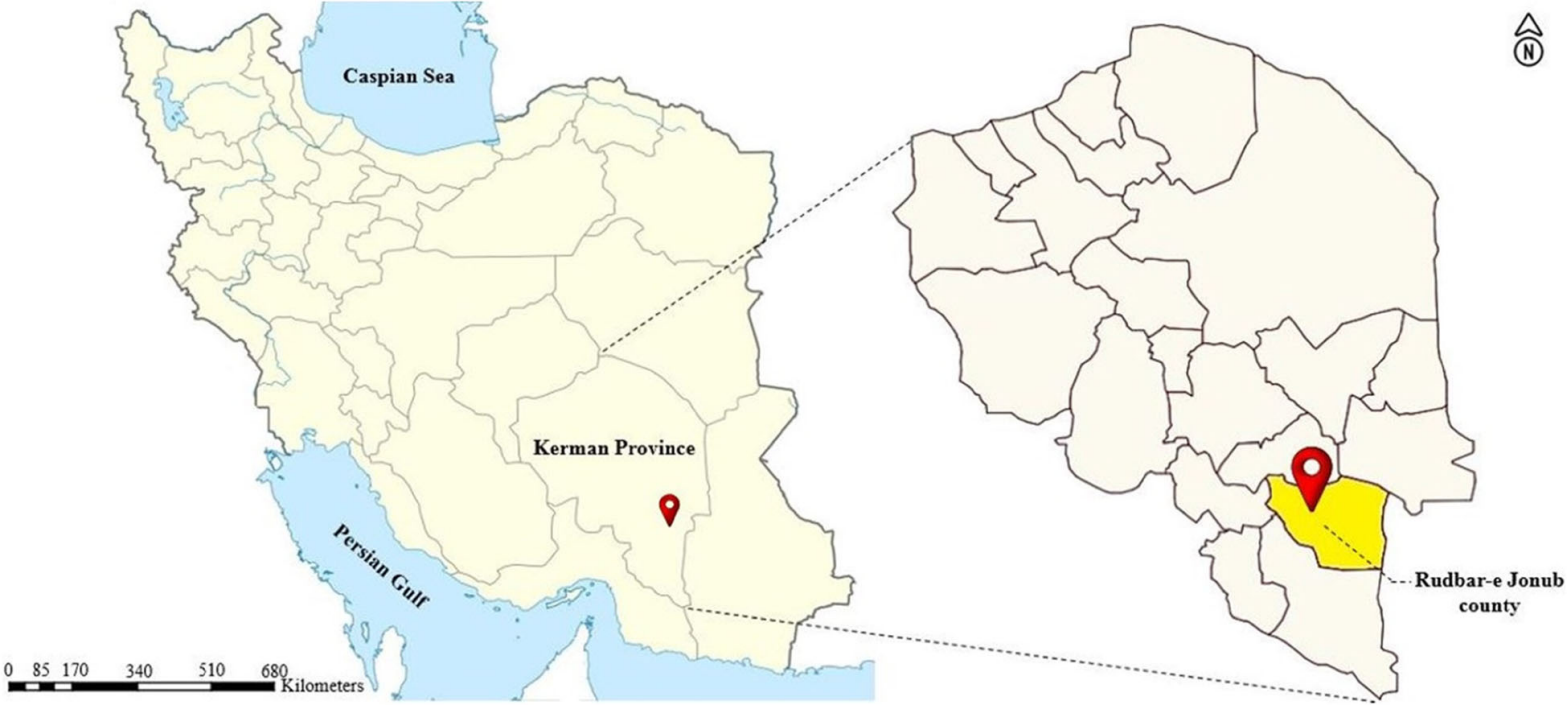
Map of the study area. Left: Map of Iran, Right: Location of Rudbar-e Jonub county in Kerman province

Rudbar-e Jonub county with an area about 7000 km^2^ located in “Hamun-e Jaz Murian” wetland basin, southeast edge of Kerman province, southeastern Iran (28°01′45.5″N 57°59′34.8″E). It is comprised of two districts and four rural districts. Based on information of the Statistical Center of Iran represented in 2016, Rudbar-e Jonub has a population of 105,992 inhabitants in 27,428 households. About 80% of the population settled in rural areas. This area has a warm and semi-arid climate (Fig. 1) [7].

### Study design

This cross-sectional study was conducted in Rudbar-e Jonub county in 2018. Eight hundred and sixty-one (861) stool specimens were collected through a multistage cluster sampling from 4 rural districts of the county as the study strata. In each rural district, health centers selected through proportional-to-size random sampling. A total of 30 health centers were selected throughout the county. Ten households covered by each health center were selected using the systematic sampling approach. All members of the selected households were invited to take part in the study. If individuals in a household refused to take part, the next household was invited. A pre-designed checklist including sex, age group, occupation, education level, source of drinking water, type of residency, and animal close contact was filled for each participant. Out of a total of 1500 individuals approached, 42.6% refused to give the sample (Response rate = 57.4%).

### Sample collection and laboratory analysis

Stool specimens were collected in the pre-labeled, wide-mouth, plastic containers. At the laboratory section of the health centers after examining the specimens for consistency, color, the presence of blood, mucus and adult intestinal helminths, macroscopically, a direct wet-mount was prepared and examined for the fresh specimens with a watery consistency or containing blood or mucus under low-power objective (10×) and high dry objective (40×) for suspicious objects. Also, a part of each collected specimen (approximately 2 g) was cultured on agar plates. Then, all specimens were preserved in 10% buffered formalin and transported to the laboratory affiliated to Jiroft University of Medical Sciences for formalin-ethyl acetate sedimentation method. Merthiolate-iodine-formaldehyde (MIF) solution was used to temporary staining of sediments obtained from the formalin-ethyl acetate method. The specimen collection, processing, shipping, and the parasitological methods were carried out as described by Garcia et al. [8]. All microscopic evaluations and identification were made by the same observer(s) blinded to participants information. Specimens were considered positive if the helminth eggs, larvae, or cysts and/or trophozoites of protozoans were detected by at least one of the three methods.

### Statistical analysis

The frequency was calculated for qualitative and categorical variables. The crude and adjusted associations between intestinal parasitic infection and determinants were assessed using univariate and multiple logistic regressions. To adjust for the population distribution, poststratification corrections were made to sampling weights. Statistical significance was accepted at *p* values < 0.05. Statistical analysis was done using Stata v.14.2 (Stata Corp LP, Texas, USA).

## Results

Over 1 year, 861 stool specimens from the Rudbar-e Jonub participants including 400 (46.1%) males and 461 (53.9%) females were collected. The majority of the participants were children up to 9 years of age (25.9%). Only 6.9% of the participants had an academic education and 20.5% of them were illiterate. 35.6% of the participants had no safe drinking water (Table 1).

**Table 1:** Socio-demographic characteristics of participants (*n* = 861)

Characteristics	Number	Percent (un-weighted)	Percent (weighted)
Sex			
Male	400	46.5	46.1
Female	461	53.5	53.9
Age group			
≤ 9	234	27.2	25.9
10 to 19	151	17.5	16.7
20 to 29	134	15.6	15.5
30 to 39	156	18.1	18.4
40 to 49	98	11.4	11.5
50≤	88	10.2	12
Occupation			
Housewife	222	25.8	26.6
Children	132	15.3	13.2
Student	202	23.5	24.2
Farmer	136	15.8	15.7
Employed	70	8.1	8.9
Unemployed	24	2.8	2.5
Others	75	8.7	8.9
Education level			
Children under 6-yr	135	15.7	13.6
Elementary school	242	28.1	28.9
High school	256	29.7	30.1
University	55	6.4	6.9
Illiterate	173	20.1	20.5
Source of drinking water			
Tap water	558	64.8	64.4
Spring or well water	303	35.2	35.6
Animal close contact			
Yes	501	58.2	60.1
No	360	41.8	39.9
Residency status			
Rural	751	87.2	87.7
Urban	110	12.8	12.3

At least one species of the intestinal parasites was found in 34.2% (95% CI 30.1 to 38.2) of the participants. Multiple infections with 2 or 3 parasitic agents constituted 22.7% of 295 infected cases. Any parasitic agent was seen in direct examination (performed on the samples with a watery consistency) and also agar plate culture method. The prevalence of detected intestinal parasites by formalin-ether sedimentation method embedded in Table 2. The prevalence of protozoan parasites 32.3% (95% CI 28.4 to 36.5) was significantly higher than helminthic parasites 3.2% (95% CI 2.1 to 4.7). *Blastocystis hominis*, *Entamoeba coli* and *Giardia lamblia* were the most common intestinal protozoan with a prevalence of 13.3 (95% CI 11.0 to 15.5), 11.4 (95% CI 8.7 to 15.4) and 10.6 (95% CI 8.5 to 13.1), respectively. *Entamoeba histolytica/dispar*, *Iodamoeba bütschlii*, *Chilomastix mesnili* and *Entamoeba hartmanni* were other detected protozoan parasites in the study. Also, the most prevalent helminthic infection was *Hymenolepis nana* with a prevalence of 2.4 (95% CI 1.5–3.9). *Ascaris lumbricoides* and *Enterobius vermicularis* were other detected helminthic parasites.

**Table 2:** Prevalence of intestinal parasites in the participants (*n* = 861)

Parasite	Number of infected	Prevalence (95%CI^a^)
Protozoa		
*Blastocystis hominis*	114	13.3 (11.0 to 15.5)
*Entamoeba coli*	98	11.4 (8.7 to 15.4)
*Giardia lamblia*	92	10.6 (8.5 to 13.1)
*Entamoeba histolytica/dispar*	14	1.5 (0.8 to 2.6)
*Iodamoeba bütschlii*	11	1.0 (0.5 to 1.9)
*Chilomastix mesnili*	5	0.5 (0.1 to 1.2)
*Entamoeba hartmanni*	4	0.4 (0.1 to 1.0)
Total^b^	280	32.3 (28.4 to 36.5)
Helminths		
*Hymenolepis nana*	22	2.4 (1.5 to 3.9)
*Enterobius vermicularis*	3	0.3 (0.0 to 0.9)
*Ascaris lumbricoides*	3	0.3 (0.1 to 1.3)
Total	28	3.2 (2.1 to 4.7)
Total^b^	295	34.2 (30.1 to 38.2)

^a^*CI* confidence interval

^b^There were also some cases of coinfection with two or three species

The results of unadjusted and adjusted logistic regression analyses of the risk factors associated with intestinal parasitic infections among the participants embedded in Table 3. Among possible risk factors investigated in this study, the source of drinking water and residency status (rural/urban) were found to have a significant association with intestinal parasitic infections (*p* value < 0.05). There was no association between the infection and sex, age group, occupation, education level, and animal close contact.

**Table 3:** Univariate and multiple analysis of intestinal parasitic infections and potential risk factors (*n* = 861)

Risk factors	Prevalence (95% CI)	OR^a^ (95% CI)	OR^b^ (95% CI)
Sex			
Male	35.4 (29.1 to 42.4)	1	1
Female	32.8 (28.1 to 37.8)	1.0 (0.7 to 1.3)	1.1 (0.8 to 1.6)
Age group			
≤ 9	42.6 (34.6 to 53.7)	1	1
10 to 19	35.9 (28.0 to 44.7)	0.8 (0.5 to 1.3)	0.7 (0.3 to 1.1)
20 to 29	23.9 (17.0 to 32.5)	0.5 (0.3 to 0.8)	0.4 (0.2 to 1.0)
30 to 39	31.1 (23.9 to 39.4)	0.7 (0.4 to 1.1)	0.8 (0.4 to 1.2)
40 to 49	36.2 (26.4 to 47.3)	0.8 (0.4 to 1.3)	0.8 (0.4 to 1.4)
50≤	25.4 (16.4 to 37.2)	0.6 (0.3 to 1.1)	0.5 (0.3 to 1.2)
Occupation			
Housewife	30.6 (24.1 to 38.0)	1	1
Children	32.4 (24.5 to 41.4)	1.0 (0.6 to 1.7)	2.2 (0.5 to 3.8)
Student	45.8 (36.0 to 56.1)	1.4 (0.9 to 2.1)	1.1 (0.5 to 2.1)
Farmer	32.3 (24.6 to 41.0)	1.1 (0.7 to 1.7)	1.0 (0.5 to 1.7)
Employed	20.3 (12.0 to 32.1)	0.6 (0.3 to 1.1)	0.6 (0.3 to 1.3)
Unemployed	47.0 (26.2 to 68.8)	1.4 (0.6 to 3.1)	1.3 (0.5 to 3.5)
Others	27.6 (17.7 to 40.5)	0.8 (0.4 to 1.4)	0.6 (0.3 to 1.2)
Education level			
High school	29.7 (22.2 to 38.4)	1	1
Children under 6-yr	38.5 (29.3 to 48.6)	0.7 (0.4 to 1.1)	0.1 (0.1 to 0.6)
Elementary school	35.1 (29.0 to 41.7)	0.9 (0.6 to 1.3)	0.7 (0.5 to 1.1)
University	22.0 (12.3 to 36.1)	0.4 (0.2 to 0.9)	0.5 (0.2 to 1.2)
Illiterate	33.0 (25.6 to 41.2)	0.8 (0.5 to 1.3)	0.7 (0.4 to 1.2)
Source of drinking water			
Tap water	31.6 (26.4 to 37.3)	1	1
Spring or well water	38.4 (32.6 to 44.6)	1.3 (1.0 to 1.8)	1.3 (1.0 to 1.8) ^*^
Animal close contact			
No	32.7 (27.6 to 38.2)	1	1
Yes	34.9 (29.4 to 40.9)	1.1 (0.8 to 1.4)	1.0 (0.7 to 1.3)
Residency status			
Rural	34.8 (30.5 to 39.3)	1	1
Urban	28.6 (20.0 to 39.0)	0.6 (0.3 to 0.9)	0.4 (0.2 to 0.7) ^*^

^a^Crude odds ratio

^b^Adjusted odds ratio

^*^Indicates *p < 0.05*

## Discussion

The results of this study showed one-third (34.2%) of the inhabitants in Rudbar-e Jonub were infected by intestinal parasites. This finding is consistent with the studies carried out in apparently healthy inhabitants in recent decades in rural and tribal areas of the country. Barkhori et al. [9] reported 28% of the infection in Jiroft district, near the studied area. Similarly, a relatively high prevalence of the infection in nomadic tribes of Khuzestan province (25.4%) and rural inhabitants of Mazandaran (25%), Kohgiluyeh and Boyer-Ahmad (37.5%), Lorestan (32.5) and Hamadan (35.1%) provinces has been reported [10–14]. Also, Hemmati et al. [15] in a study on inhabitants of Rudehen in Tehran province, capital of Iran, have reported 32.7% of the infection. Besides, some studies reported a significant prevalence of 48.8% in rural inhabitants of Hormozgan [6] and 56% in nomadic tribes of Chaharmahal and Bakhtiari provinces [4].

The finding of the current and aforementioned studies reflects the fact that in spite of advances in sanitation and personal/public health measures, it seems there is still not enough arrangements for controlling of intestinal parasites and these neglected agents especially protozoans are still a significant public health problem in rural and tribal areas of the country. The importance of this issue will become clear when we know these reported prevalences seem to be less than the actual value because in most studies on intestinal parasites prevalence only one stool specimen of subjects was collected for examination whereas for a standard diagnosis collecting three sequential specimens in three alternate days is required [16]. Also, in most studies, no specific methods such as modified acid-fast staining and Graham test for diagnosis of coccidia and *E. vermicularis* respectively, are performed.

*Blastocystis sp.*, *E. coli*, and *G. lamblia* were the most common intestinal parasites among the study population similar to other studies in Iran [6, 9–11]. All of them have a fecal-oral transmission mode, indicates poor hygiene in Rudbar-e Jonub county. According to the current findings, the prevalence of *Blastocystis sp.* infection was found at 13.3% (95% CI 11.0 to 15.5). The reported range of *Blastocystis* infection in the apparently healthy populations in the country varies from 7.5 to 28.4% [10, 15]. Several studies have revealed an association between carrying the parasite and some clinical manifestations that is controversial yet [17–19]. In this study, the highest prevalence of *Blastocystis* infections was observed in the age group of 30 to 39 year. Some studies have suggested that the incidence of *Blastocystis* infection increases with age [15, 20]. Due to the unclear aspects of zoonotic, mode of transmission, and potential host factors important for colonization, it seems, discussion about such association needs more evidence.

The prevalence of *E. coli* in the current study was found 11.4% (95% CI 8.7 to 15.4). In the studies carried out in recent decade on apparently healthy people in Iran, the prevalence of *E. coli* reported up to 18.9% [13]. Although *E. coli* and other non-pathogenic parasites detected in this study do not cause infection, their presence indicates the fecal-oral transmission in the host, which is an indicator for the general assessment of the hygiene status of the area. In this study, the prevalence of *Giardia* infection was 10.6% (95% CI 8.5–13.1).

The prevalence of *G. lamblia* in the apparently healthy populations in Iran has been reported 33.9 and 10.2% in 2008 [10, 11], 28.2 and 8.9% in 2009 [4, 14], 17.2% in 2011 [6], 2.2% in 2014 [13], 17.4 and 7.8% in 2016 [9, 12], and 1.2% in 2017 [15]. The overall prevalence of *Giardia* has shown a declining trend during the past decade but it appears to be still relatively high depending on the target population.

The prevalence of human helminthic diseases declined sharply in recent decades throughout Iran but some of them, particularly those with direct fecal-oral transmissions, such as *Hymenolepis* and *Enterobius*, remain common in some parts of the country [21]. In the current study, the helminthic infection was limited to only three species among which most infection was related to *H. nana* 2.4% (95% CI 1.5 to 3.9). Although the prevalence of *H. nana* in human has fallen since 1970 [21] it remains relatively common in the rural an tribal areas of Iran [4, 9, 10]. *E. vermicularis* with a 0.3% (95% CI 0.0 to 0.9) prevalence was another detected helminthic infection. Given that the Graham test was not done in this study, the actual prevalence is probably higher than the reported value. Reduction in the prevalence of these parasites will need more direct interventions, such as the employing experienced technician in medical laboratories and treatment of infected cases and also, health education to informing people from transmission route of these helminthic infections. Also, 0.3% (95% CI 0.1 to 1.3) of infection with *A. lumbricoides* as a soil-transmitted helminth (STH) was detected in this study. Due to effective measures to improve public health in Iran the prevalence of *A. lumbricoides* dropped from 46.7% in 1987 [22], 17.8% in 1992 [23], and 16.3% in 1996 [24] to 0.4% in this study. Low prevalence of intestinal helminthic infections in Rudbar-e Jonub is in concordance with the result of recent studies in other parts of Iran [9, 13, 15].

In this study, several possible determinants associated with intestinal parasitic infections were investigated and a significant association was found between intestinal parasitic infections and, source of drinking water and residency status (rural/urban). The source of drinking water is an important risk factor for infection with intestinal protozoa such that a waterborne transmission of all detected protozoa in this study is possible. In this study, 35.2% of the participants were deprived of safe drinking water. Prevalence of intestinal parasites in participants who used non-sanitary drinking water was 38.4% (95% CI 32.6 to 44.6%), significantly higher than other participants, suggesting the possibility of waterborne transmission. Similar results have been reported in some studies regarding the importance of sources of drinking water [9, 15]. The need for improvement of public health infrastructure in Rudbar-e Jonub county is evident. About 80% of the population in Rudbar-e Jonub have a rural lifestyle. In this study, the prevalence of intestinal parasites was higher in rural areas than in urban areas, significantly. Rural lifestyle is itself risky due to insufficient infrastructure, disorganization in health services and lower socioeconomic living conditions [25]. Several studies on human parasitic infections have revealed a common association between parasitic infections and lower socioeconomic status of rural area in Iran [5, 6, 26].

In the current study, intestinal parasitic infections showed no significant association with sex, age group, occupation, education level, and animal close contact. In rural life of Rudbar-e Jonub, most women are involved in outdoor activities including farming and animal husbandry as like as men which exposes them to infection as much as men. Also, in this county, most housewives and students are involved in farming and animal husbandry and the occupational variation is low. Therefore, it is complex to discuss the association between sex and occupation with intestinal parasitic infections in this area. According to current finding, there was no significant association between sex and occupation with the infection, similar most studies carried out on the prevalence of intestinal parasites [9–11, 15].

The prevalence of parasitic infections in the age group ≤9 year was 42.6% (95% CI 34.6 to 53–7%), higher than other groups but there was no statistically significant association between age groups and parasitic infections. The most common intestinal parasite in this age group was *G. lamblia*. Univariate analysis showed a significant association between *Giardia* infection and age group. It seems the lower levels of personal hygiene in children can be attributed to the higher prevalence of intestinal parasites in them. Regarding the participant’s education level, although no significant association was found between the prevalence of intestinal parasitic infections and level of education, the results of this study indicate that as the level of literacy increases, the rate of parasitic infection decreases. Educated people are more aware of the transmission of parasitic infection and they may apply the necessary measurements to avoid the infection. Also, the results of this study showed the odds ratio of the infection in participants with and without close contact with the animal was almost same. These results indicate that domestic animals do not play a bold role in the transmission of intestinal parasites detected in this study to humans in Rudbar-e Jonub county.

### Limitations

Because of cultural reasons, many households were reluctant to give their specimens which led to a low response rate (57.4%) and also, stool specimens were collected once from each participant whereas for standard diagnosis of intestinal parasites, at least three specimens in three alternate days are necessary and also Graham test for *E. vermicularis* diagnosis was not done. Furthermore, because of financial constraints and limited facilities, molecular methods for identification of *E. histolytica/dispar* complex was not done. Another limitation of this study was the lack of collecting information on clinical symptoms.

## Conclusions

This study revealed a high prevalence of intestinal protozoan infections among inhabitants of Rudbar-e Jonub county. Despite the downtrend of parasitic infections in Iran, compared to past decades, intestinal parasites especially protozoans remain a challenging public health problem wherever sanitation and health measures are limited.

## Data Availability

The datasets generated and/or analyzed during the current study may be made available from the corresponding authors on reasonable request.

## Abbreviations

CI: Confidence interval
MIF: Merthiolate-iodine-formaldehyde
OR: Odds ratio
STH: Soil-transmitted helminth
